# Intestinal helminth co-infection and associated factors among tuberculosis patients in Arba Minch, Ethiopia

**DOI:** 10.1186/s12879-017-2195-1

**Published:** 2017-01-13

**Authors:** Getaneh Alemu, Mohammedaman Mama

**Affiliations:** Department of Medical Laboratory Science, Arba Minch University, Arba Minch, Ethiopia

**Keywords:** Helminth, Tuberculosis, Co-infection

## Abstract

**Background:**

Helminths affect the outcome of tuberculosis by shifting cell mediated immune response to humoral and by total suppression of the host immune system. On the reverse, *Mycobacterium* infection favors immune escape of helminths. Therefore assessing helminth co-infection rate and predisposing factors in tuberculosis patients is mandatory to set strategies for better case management.

**Methods:**

Facility based cross-sectional study was conducted in Arba Minch to assess the prevalence and associated factors of intestinal helminths among pulmonary tuberculosis patients from January to August, 2016. A structured questionnaire was used to capture data about socio-demographic characteristics, clinical history and possible risk factors for intestinal helminth infections. Height and weight were measured to calculate body-mass index. Appropriate amount of stool was collected and processed by direct saline and formol-ether concentration techniques following standard protocols. All the data were analyzed using SPSS version 20.0.

**Results:**

A total of 213 (57.3% male and 42.7% female) pulmonary tuberculosis patients were participated in the study. The overall co-infection rate of intestinal parasites was 26.3%. The infection rate of intestinal helminths account 24.4% and that of intestinal protozoa was 6.1%. *Ascaris lumbricoides* accounted the highest frequency of 11.3%. Living in rural residence (AOR = 3.175, 95% CI: 1.102–9.153, *p* = 0.032), Eating vegetables/ fruits without washing or peeling off (AOR = 2.208, 95% CI: 1.030–4.733, *p* = 0.042) and having body-mass index <18.5 (AOR = 3.511, 95% CI: 1.646–7.489, *p* = 0.001) were associated with intestinal helminth infection.

**Conclusion:**

The infection rate by intestinal helminths was 24.4%. *Ascaris lumbricoides* was the most prevalent helminth. Residence, habit of washing vegetables/fruits before use and body-mass index were associated factors with intestinal helminthiasis. Therefore health care providers should screen and treat TB patients for intestinal helminthiasis in order to ensure good prognosis.

## Background

Tuberculosis (TB) remains one of the world’s major public health problems [1, 2]. Even if TB is slowly declining each year since 2000, death from the disease is still unacceptably high [1]. According to World Health Organization (WHO) report, about 10.4 million people developed TB and 1.4 million died from all forms of TB in 2015 globally. One quarter of the cases were in the African Region. Ethiopia is ranked 8^th^ and 2^nd^ among 22 TB-high burden countries of the World and Africa respectively. The incidence and mortality (excluding HIV related deaths) because of all forms of TB were 192 and 26/100,000 population, respectively in 2015. It remains one of the leading causes of mortality due to communicable diseases in Ethiopia [1].

Control of *Mycobacterium tuberculosis* (*M. tuberculosis*) infection is mainly dependent on the success of the interaction between innate and adaptive immune responses of the host. The cell mediated immunity mainly determines the disease outcome [3, 4]. In most immuno-competent individuals, infected macrophages interact with both CD4+ and CD8+ T cells and control the infection mainly via T helper(Th) -1 type inflammatory response [4, 5]. Individuals whose immunity is suppressed or shifted toTh2 type are more susceptible to develop active infection and more acute morbidity. Malnutrition, diabetes mellitus, HIV, helminth infection, different forms of cancer and prolonged use of steroid drugs are among the factors which suppress/shift the immune response [4–6]. Helminths induce a strong Th2-type immune response characterized by production of cytokines like interleukin (IL) - 4, IL-5, IL-9, and IL-13 and increased levels of circulating IgE antibodies and eosinophils [4]. Prolonged Th2 response is followed by activation and expansion of both natural and inducible regulatory T cells [6, 7]. All these immune-modulations favor survival, multiplication and dissemination of *M. tuberculosis* to develop active TB and the associated sequelae [8, 9]. Such independent immune-modulation by both helminths and *M. tuberculosis* determines the pathogenesis and outcome of both infections [10].

The geographical distribution of intestinal helminth infections and TB overlap substantially that co-infection in humans has been a major emerging public health problem in poorest regions of the world [3, 4, 11]. Strong association between active pulmonary TB (PTB) and helminthiasis has been shown from previous studies [3, 11, 12]. However the helminth species and rate of co-infection with PTB vary from place to place. Climate conditions, socio-demographic characteristics and living standards of the population are believed to determine the type of helminths existing in various TB endemic areas. This necessitates sufficient data to be collected about the co-infection rate and common co-infecting helminth species at different geographical settings; because the data will be an input for policy makers to modify TB case management protocols at local level based on the existing conditions. However very limited data is available in Ethiopia that is generated only from Gondar, Northwest Ethiopia [2–4, 13]. As to the best of our knowledge, no similar study has been conducted in southern Ethiopia so far.

On the other hand, the clinical significance and associated risk factors of helminths is more related to, and extensively studied in children. This is because of considering asymptomatic or sub-clinical level of morbidity in adults. However in adults with concurrent chronic diseases, helminthes behave aggressively to result in severe morbidity and even death [2]. Despite this, data about the infection rate and associated risk factors among adults is lacking in the study area. Therefore in this study, we have assessed intestinal helminth co-infection rate and associated factors among PTB patients in Southwest Ethiopia.

## Methods

### Study design and area

Facility based cross-sectional study was conducted in selected TB clinics of Arba Minch town and Arba Minch zuria district from January to August, 2016. Arba Minch is located 454 kms south of Addis Ababa. It is found at an altitude of 1200–1300 m above sea level with an average annual temperature of 29.7 °C and rain fall of 900 mm [14]. In the town, there is one hospital and two health centers. All the three institutions have TB diagnosis laboratories and treatment clinics. Arba Minch zuria woreda is situated at average of 1285 m above sea level surrounding Arba Minch town [14]. There are seven health centers in the woreda giving routine health services including TB diagnosis and treatment.

### Selection of study participants

Information about PTB patient flow at each governmental health institutions of Arba Minch town and surrounding rural district was collected first. Relatively, Arba Minch Hospital, two health centers in the town (Arba Minch health center and Shecha health center) and one health center in Arba Minch zuria district (Shelle health center) were found to be with high PTB patient flow. Therefore, TB clinics in the above mentioned health facilities were purposively selected for data collection. Consecutive newly diagnosed and on treatment PTB patients who attended TB clinics during the study period and have given informed written consent were included in the study. Only PTB patients diagnosed based on the national diagnosis guideline and 15–65 years of age were recruited. Cases are diagnosed to be PTB patients if there is persistent (for ≥2 weeks) cough two sputum examinations reveal positive result for acid fast bacilli or one sputum examination is positive and chest x-ray result is suggestive for PTB.

Pregnant mothers, patients who have taken anti-helminth drug within three months before data collection and severely ill patients who are unable to respond to research questions were excluded from the study. Patients with other immunosuppressive chronic diseases were excluded by referring to their medical records. Under 15 age groups were also excluded aiming to assess intestinal helminth prevalence and associated factors among adolescents and adults. Patients above 65 years of age were not involved in the study as their immune response is expected to be weakened naturally.

### Data collection

#### Socio-demographic characteristics

Nurses who are fluent speakers in the local language (Gamo) were trained for data collection. A pretested structured questionnaire administered through face to face interview was used to collect data about socio-demographic characteristics and associated factors for intestinal helminth infection after translating to the local language. Clinical data was also collected by interview and from the patients’ log book.

### Anthropometric measures

Body-mass index (BMI) is considered as the most suitable, objective anthropometric indicator of nutritional status of adults. To calculate BMI, measurements of height and weight were under taken by trained nurses following a standard procedure explained elsewhere [15]. According to the WHO definition, individuals with BMI < 18.5 are malnourished and those with BMI < 16 are severely malnourished [16].

### Laboratory methods

An appropriate amount of stool sample was collected and delivered to Parasitology laboratories of Arba Minch hospital or Arba Minch University, college of medicine and health sciences teaching laboratory following standard collection and transportation protocols [17]. About 50 mg of stool was processed immediately after collection using direct saline method in order to identify motile trophozoites of intestinal protozoa and larvae of *S. stercoralis*. One or two drops of normal saline were mixed with an approximate of 50 mg stool on a clean slide. A uniform suspension was made using an applicator stick and covered with a clean cover slip. The entire preparation was systematically screened using the 10× and 40× objective lenses for detection of protozoa trophozoites and cysts as well as helminth ova and larvae. The remaining stool was processed by the formol-ether concentration technique, which is considered as the most sensitive for most intestinal helminths and protozoan cysts. About 1 gm of stool was added to a clean 15 ml conical test tube containing 7 ml of 10% formal saline. The stool was gently suspended with the formal saline using applicator stick. The suspension was filtered through a sieve in to a second centrifuge tube. After adding 3 ml of diethyl ether, contents in the second tube were centrifuged at medium speed (2500 rpm) for 5 min. The supernatant was poured off and smear on a clean slide was prepared from the sediment and covered with clean cover slip. The preparation was examined in the same way as that of the direct saline method. Negative results were reported after assessing the whole smear under10 x objective. Investigators supervised all aspects of data collection and laboratory procedures.

### Statistical analysis

Data was edited, cleaned, entered and analysed using SPSS version 20.0. Descriptive statistics are calculated to describe the study population characteristics. Bivariate logistic regression is used to assess associations between categorical variables. Multivariate regression model then followed for variables with *p* ≤ 0.25 in the bivariate analysis. Association between variables was considered statistically significant only if *P*-value <0.05 at 95% confidence level.

## Results

### Socio-demographic and clinical data

A total of 213 PTB patients participated in the study. Eighty six of them were from Arba Minch hospital while 69, 22 and 36 participants were recruited from Arba Minch, shecha and shellehealth centres respectively. One hundred twenty-two (57.3%) were male and 91 (42.7%) were female participants. The highest number of participants, 136 (31%), belong to the age group 15–34 years old while the lowest number, 7 (3.3%), belongs to ≥55 years of age. Majority (92.0%) of the study participants commonly cook their food at home. Among those having frequent habit of eating raw vegetables/fruits, 136 (66.3%) wash or peel off before use. Most respondents, 204 (95.8%) and 210 (98.6%), often wash hands before meal and defecation respectively (Table 1).

**Table 1:** Socio-demographic characteristics of TB patients attending selected health institutions of Arba Minch town and Arba Minch zuria district from January to August, 2016

Variables		Frequency		
		Number (n)	Percent (%)	Total no. of respondents
Sex	Male	122	57.3	213
	Female	91	42.7	
Age group	15–34	136	63.8	213
	35–54	70	32.9	
	55–65	7	3.3	
Marital status	Single	115	54	213
	Married	98	46	
Educational level	Illiterate	60	28.2	213
	Primary and above	153	71.8	
Residence	Urban	166	77.9	213
	Rural	47	22.1	
Have/use latrine	Yes	182	85.4	213
	No	31	14.6	
<14 children live in the house	Yes	64	30	213
	No	149	70	
Common food source	Cooked at home	196	92.0	213
	Hotel	17	8.0	
Wash raw vegetables/fruits before use	Often or always	136	66.3	205
	Never or occasionally	69	33.7	
Habit of eating raw meat	Often or always	176	82.6	213
	Never or occasionally	37	17.4	
Hand washing habit before meal	Often or always	204	95.8	213
	Never or occasionally	9	4.2	
Hand washing habit after defecation	Often or always	210	98.6	213
	Never or occasionally	3	1.4	
Water source for washing utensils	Pipe	171	80.3	213
	River/lake/stream	42	19.7	
Water source for bathing	Pipe	166	77.9	213
	River/lake/stream	47	22.1	
Water source for drinking	Pipe	199	93.4	213
	River/lake/stream	14	6.5	
Swimming habit	Yes	37	17.5	212
	No	175	82.5	
Shoe wearing habit	Yes	202	94.8	213
	No	11	5.2	
Raise cattle	Yes	63	29.6	213
	No	150	70.4	
Raise pets (cat/dog)	Yes	73	34.3	213
	No	140	65.7	
Use night soil for farming	Yes	32	15.0	213
	No	181	85.0	

Out of 213 participants, 24 (11.3%) had self-reported diarrhea within the last 3 months before this data was collected. Ten of them contracted the last diarrheal episode within the last two weeks. Two hundred nine (98.1%) were tested for HIV of whom 20 (9.6%) were positive. About one third (33.3%) were with BMI <18.5. During the time of interview, 200 (93.9%) of the participants have already started anti-TB treatment while the rest 13 (6.1%) were contacted as soon as diagnosed before the start of treatment (Table 2).

**Table 2:** Clinical history of TB patients attending selected health institutions of Arba Minch town and Arba Minch zuria district from January to August, 2016

Variables		Frequency		
		Number (n)	Percent (%)	Total no. of respondents
History of diarrheal within 3 months	Yes	24	11.3	213
	No	189	88.7	
Time of last diarrhoea occurrence	Before a month	11		24
	Before 2 weeks	3		
	Within 2 weeks	10		
Duration of last diarrhoea occurrence	> a month	2		24
	< a month	13		
	< a week	9		
Other GIT discomfort within 3 months	Yes	40	18.8	213
	No	173	81.2	
Started anti-TB treatment	Yes	200	93.9	213
	No	13	6.1	
Course of treatment	First	190	95.0	200
	Re-treatment	10	5.0	
Duration of treatment in months	<2	45	22.5	200
	2–4	55	27.5	
	>4	100	50.0	
Started anti-TB treatment	Yes	200	93.9	213
	No	13	6.9	
HIV status	Positive	20	9.6	209
	Negative	189	90.4	
BMI	<18.5	71	33.3	213
	≥18.5	142	66.7	

### Prevalence of parasites

The overall co-infection rate of intestinal parasites was 26.3%. The prevalence of intestinal helminths account 24.4% and that of intestinal protozoans was 6.1%. Totally six helminths were detected among which *Ascaris lumbricoides (A. lumbricoides)* was with the highest frequency (24, 11.3%) followed by hook worms (18, 8.5%). *Giardia lamblia (G. lamblia)* and *Entamoeba histolytica* (*E. histolytica)* were the only intestinal protozoans detected from 10 (4.7%) and 4 (1.9%) participants respectively (Table 3).

**Table 3:** Prevalence of intestinal parasites in TB patients attending selected health institutions of Arba Minch town and Arba Minch zuria district from January to August, 2016

Parasites		Frequency	
		Number (n)	Percent (%)
Helminths	*A. lumbricoides*	24	11.3
	*Hook worm*	18	8.5
	*T. trichiura*	9	4.2
	*Taenia species*	5	2.3
	*H. nana*	2	0.9
	*S. stercoralis*	2	0.9
Number of helminth species per participant	1	40	18.8
	2	10	4.7
Protozoa	*G. lamblia*	10	4.7
	*E. histolytica*	4	1.9
Total prevalence	Helminths	52	24.4
	Protozoa	13	6.1
	Parasite (helminth + protozoa)	56	26.3

### Factors associated with intestinal helminth co-infection

Residence had significant association with intestinal helminth infection (*p* = 0.032) that rural residents were 3 times more likely (AOR = 3.175, 95% CI: 1.102–9.153) to be infected than urban dwellers. According to the bivariate analysis model, respondents who commonly cook their food at home were 3 times more affected (95% CI: 1.119–8.429, *p* = 0.029) than those who commonly get their food from hotel; but in the multi-variate analysis, food source was not associated with intestinal helminthiasis. Eating vegetables/ fruits without washing or peeling off was also significantly associated with helminthiasis (*p* = 0.042). The odds of infection were 2 times higher (AOR = 2.208, 95% CI: 1.030–4.733) for those eating vegetables/ fruits without washing or peeling off as compared to participants who wash or peel off before eating fruits/vegetables. Participants who often walk bare foot were at higher risk to helminth infection (COR = 2.27, 95% CI: 1.168–4.413, *p* = 0.016) but this association was not significant when adjusted for confounding factors. Nutritional status had significant association with intestinal helminthiasis (*p* = 0.001) that participants with BMI <18.5 were 3.5 times at higher risk (AOR = 3.511, 95% CI: 1.646–7.489) of acquiring helminth infection as compared to those with ≥ 18.5. The other considered socio-demographic and clinical factors didn’t show significant level of association with intestinal helminth infection (Tables 4 and 5).

**Table 4:** Factors associated with intestinal helminth co-infection among TB patients attending selected health institutions of Arba Minch town and Arba Minch zuria district from January to August, 2016

Variable	Number examined	Rate of helminth infection (%)	Crude OR (95% CI)	*P* value	Adjusted OR	*P* value
Sex						
Male	122	32 (2.6%)	1.085 (0.578–2.037)	0.800		
Female	91	24 (2.6%)				
Age						
15–34	136	32 (23.5%)		0.700		
35–54	70	19 (27.1%)	0.826 (0.427–1.527)			
55–65	7	1 (14.3%)	1.846 (0.214–15.909)			
Educational status						
Illiterate	60	13 (21.7%)	1.416 (0.684–2.633)	0.349		
Primary/above	153	43 (28.1%)				
Marital status						
Single	115	29 (25.2%)	1.116 (0.597–2.088)	0.731		
Married	98	27 (27.6%)				
Residence						
Urban	166	46 (27.7%)	2.619 (1.042–6.584)	0.041	3.175 (1.102–9.153)	0.032
Rural	47	6 (12.8%)				
Have/use latrine						
Yes	182	41 (22.5%)	0.529 (0.234–1.193)	0.125		
No	31	11 (35.5%)				
Common food source						
Cooked at home	196	44 (22.4%)	3.071 (1.119–8.429)	0.029	2.866 (0.900–9.131)	0.075
Hotel	17	8 (47.1%)				
Habit of washing vegetables/fruits						
Yes	141	26 (18.4%)	2.835 (1.468–5.476)	0.002	2.208 (1.030–4.733)	0.042
No	64	25 (39.1%)				
Habit of eating raw meat						
Often or always	176	43 (24.4%)	0.994 (0.435–2.271)	0.989		
Never or occasionally	37	9 (24.3%)				
Hand washing habit before meal						
Often or always	204	48 (23.5%)	0.385 (0.099–1.490)	0.167		
Never or occasionally	9	4 (44.4%)				
Hand washing habit after defecation						
Often or always	191	40 (20.9%)	0.642 (0.057–7.222)	0.719		
Never or occasionally	22	12 (54.5%)				
Water source for drinking						
Pipe water	199	47 (23.6%)	0.433 (0.131–1.428)	0.388		
River/stream/lake	14	5 (35.7%)				
Swimming habit						
Often or always	37	16 (43.2%)	0.617 (0.285–1.338)	0.222		
Never or occasionally	175	36 (20.6%)				
Shoe wearing habit						
Often/always	155	31 (20.0%)	2.27 (1.168–4.413)	0.016	1.225 (0.238–6.297)	0.808
Never/occasionally	58	21 (36.2%)				
Raise cattle						
Yes	63	14 (22.2%)	1.187 (0.590–2.388)	0.630		
No	150	38 (25.3%)				
Raise pets (cat/dog)						
Yes	73	13 (17.8%)	1.782 (0.881–3.604)	0.108		
No	140	39 (27.9%)				
Use night soil for farming						
Yes	32	7 (21.9%)	1.182 (0.479–2.916)	0.717		
No	181	45 (24.9%)

**Table 5:** Clinical data associated with intestinal helminth co-infection among TB patients attending health institutions of Arba Minch town and Arba Minch zuria district from January to August, 2016

Variable	Number examined	Rate of helminth infection (%)	Crude OR (95% CI)	*P* value	Adjusted OR	*P* value
BMI						
< 18.5	71	25 (35.2%)	2.315 (1.217–4.401)	0.010	3.511 (1.646–7.489)	0.001
≥ 18.5	142	27 (19.0%)				
Started anti-TB treatment						
Yes	200	50 (25.0%)	1.833 (0.393–8.554)	0.441		
No	13	2 (15.4%)				
Duration of anti-TB treatment						
1–2 months	45	10 (22.2%)	1.230 (0.535–2.828)	0.885		
2–4 months	55	14 (25.5%)	1.029 (0.484–2.186)			
> 4 months						
Course of treatment						
First	190	46 (24.2%)	0.479 (0.130–1.772)	0.270		
Re-treatment	10	4 (40.0%)				
HIV status						
Positive	20	3 (15.0%)	1.929 (0.546–6.871)	0.110		
Negative	189	48 (25.4%)				

## Discussion

The co-infection rate of intestinal parasites among PTB patients in this study was 26.3% (95%CI: 20.2–32.4); higher than co-infection rate of 7.3% in China [11]. Variations in socio demographic characteristics and level of awareness about intestinal parasite transmission and prevention might be important determinants for low parasite prevalence in China as compared to the present study. Our findings show lower intestinal parasite prevalence as compared to cross-sectional study results from Gondar, Northwest Ethiopia by Alemayehu et al. (33.3%) [13] and Afework et al. (40.5%) [2]. Alemayehu et al. [13] conducted the study only among newly diagnosed PTB patients before starting treatment. Hence study population difference may be one factor for differences in intestinal parasite co-infection rate. Currently school based deworming for STH and schistosomiasis is being practiced bi-annually throughout Ethiopia which might also decrease the current transmission and prevalence of helminths.

In the present study, the total intestinal helminth co-infection rate at least with one helminth was 24.4% (95%CI: 18.8-30.5). This goes in line with findings from Brazil (27.5%) [12] and Northwest Ethiopia (29%) [4]. Lower intestinal helminth co-infection rate (7%) was reported from China [11]. Elias et al. (71%) [3] and Eba et al. (36.8%) [18], both fromNorthwest Ethiopia, have reported higher infection rate of helminthes than that of the present study. The laboratory protocol followed and differences in sample size may justify for those differences. In both previous studies, 3 stool samples were collected and examined from each participant before ruling out intestinal helminthiasis. However, in the present study, we have collected and examined a single stool specimen for the sake of logistic problems. Our sample size was 213 which is much smaller than that of Eba et al. (424) [18]. The other possible reasons for helminth co-infection rate might be due to the health extension program. More than 30 thousand rural health extension workers were trained and currently have been working at community level all over Ethiopia aiming to implement preventive health packages [19]. Creating awareness about latrine construction and utilization as well as keeping personal and environmental hygiene among the community is one of the priority concerns of the program. This substantially decreases prevalence of intestinal helminths.

*Ascaris lumbricoides* is the most prevalent helminth in Ethiopia with one third of the population being infected [20, 21]. Our findings support the existing literature that *A. lumbricoides* causes the highest co-infection rate of 11.3% among PTB patients followed by hook worms (8.5%) and *T. trichiura* (4.2%). Previous cross-sectional surveys in Ethiopia also show similar findings [3, 4, 18]. The proportion of helminth species prevalent among PTB patients varies according to a few other studies. *S. stercoralis* (72.7%) was with the highest prevalence in a survey from Brazil [12]. Hook worms were the most frequent helminths in China and Northwest Ethiopia with rates of 4.3% and 11.1% respectively [11, 13]. A stool survey from adult outpatient attendants in Kenya also revealed highest prevalence of hook worms (13.1%) among intestinal helminthes [22]. Variation in the relative distribution of helminth species based on environmental factors might be a reason for such differences. In rural areas where majority of people walk bare foot, hook worms may be more prevalent than *A. lumbricoides*. Xin et al. [23] and Alemayehu et al. [13] reported walking bare foot is associated with helminth infection. May be because most respondents are urban residents, shoe wearing habit was not associated factor in the present study.

In the present study we have shown factors associated with intestinal helminthiasis among PTB patients. The findings can help to estimate to the general adolescent/adult population of the study area as TB patients are part of the population. Xin et al. from China reported that females are 2.05 times (95% CI = 1.01–4.17) more likely to acquire intestinal parasite infections than males [23]. Females were also at higher risk according to findings in Kenya by Anderek et al. [22]. Age and sex were not significantly associated with intestinal helminth infection in this study. This finding is in line with studies conducted in China and Ethiopia [11, 13]. Variable occupational exposure status at different social settings might bring differences in exposure rate by sex.

The multivariate regression model in the present study revealed that rural residence, frequent eating of raw vegetables /fruits without washing or peeling off and BMI <18.5 are risk factors for helminth infection in PTB patients. Rural residents were 3.175 times more at risk than urban residents. Alemayehu et al. also reported similar finding [13]. The rural community is less likely to adopt life style changes that would reduce infection risk. Infective helminth ova are ingested with contaminated food or water to initiate infection that individuals often feeding on raw vegetables/fruits without washing or peeling off are more likely to be infected.

Nutrition screening, assessment and management are integral components of TB treatment and care. Therefore WHO recommended that patients with TB should be nutritionally assessed and receive nutritional care and support [24]. In this study, 33.3% of the participants were malnourished among which 35.2% were co-infected with intestinal helminths. Dodor reported 51% prevalence of malnutrition among PTB patients prior to treatment in Ghana [25]. Similarly Anurag et al. reported that 80% of women and 67% of men TB patients had moderate to severe under-nutrition of which 52% had stunting indicating chronic under-nutrition [26]. Both patient groups who started anti-TB drug or not were recruited in the present study that drugs and associated health services might play a role for lower prevalence of under nutrition. Patients with BMI < 18.5 were 3.511 (95% CI = 1.646–7.489) times at higher risk of acquiring helminth co-infection. Findings from China go in line with our results [11, 23]. This is expected as helminths cause under-nutrition [27, 28]. We had got no significant association between anti-TB treatment and its duration with helminth co-infection similarly with studies from China [11, 23]. This is unexpected and needs further large scale study accompanying effects of anti-TB drugs on host immune profile. As a limitation, immunological parameters and worm load were not assessed so that their role on TB pathogenesis could be justified.

## Conclusion

The co-infection rate of intestinal parasitic infection among PTB patients was 26.3% and that of intestinal helminths only was 24.4% in Arba Minch. *A. lumbricoides* was the most prevalent helminth with infection rate of 11.3%. Rural residence, frequent habit of eating raw vegetables/fruits without washing or peeling off and malnutrition (BMI < 18.5) were associated factors with intestinal helminth co-infection among PTB patients. We recommend to health care providers to screen and treat TB patients for intestinal helminthiasis. Health education about transmission and prevention of intestinal parasites should also be part of TB case management to ensure good TB prognosis.

## Abbreviations

AOR: Adjusted odds ratio
BMI: Body-mass index
CI: Confidence interval
HIV: Human immunodeficiency virus
IL: Interleukin
PTB: Pulmonary tuberculosis
STH: Soil transmitted helminths
TB: Tuberculosis
Th: T helper
WHO: World Health Organization
